# Observations of dialysis events in a tertiary care hospital outpatient dialysis unit over an eight-month period and significant measures implemented to reduce them

**DOI:** 10.1017/ash.2025.150

**Published:** 2025-09-03

**Authors:** Navin Kumar

## Abstract

**Introduction:** A common form of Renal Replacement Therapy is haemodialysis. Haemodialysis (HD) patients require a vascular access. Because of Frequent hospitalization the HD patients are at higher risk of developing infections. Positive Blood culture, IV antimicrobial use and signs of inflammation at vascular access site are the three dialysis events that can cause substantial morbidity and mortality in patients.The objective is to identify and implement strategies to prevent dialysis events within the facility by providing appropriate analyses of dialysis events. **Methodology:** A prospective surveillance study was performed between April’23 and November’23 at our outpatient HD facility. All HD patients were eligible for the study if they received HD on first two working days of the month. We conducted a pre-stage study for two months from April’23 to May’23 and collected data. After detailed analysis, implementation measures were included in month of June’23. The surveillance was regarded as a process improvement project and further data for dialysis events were collected till month of November’23. **Interventions:** The following interventions were adopted as process improvement in hemodialysis unit; 1). Revision of the current antimicrobial policy of dialysis unit 2). Implementation of Core interventions to prevent the dialysis event like hand hygiene observation, catheter/vascular access care observation, staff education, patient education, catheter removal, CHG for skin preparation, Catheter hub disinfection and regular surveillance with feedback of Dialysis events. 3). Revised policy for regular RO water plant disinfection and microbiological testing **Results:** 755 patients were reviewed for dialysis events during the 09-month study period. A total of 16 dialysis events were reported with overall dialysis events rates was - 2.09/100 patient-months. The rate of IV antimicrobial use was-1.19/100 patient-months and the positive blood culture rate was-0.92/100 patient-months Gram-negative bacilli were predominant in patients with central lines (n = 9); however, skin commensals and gram negative bacilli were also identified in patients with fistula or graft (n = 2). A reduction in dialysis events from 3.3 /100 patient days to 1.08/100 patient days was observed after the implementation of core interventions. **Conclusion:** Dialysis events were significantly more frequent in patients with tunnelled or non-tunnelled central venous lines compared to those with fistula or graft. In haemodialysis patients, good compliance with antimicrobial policy and regular monitoring of core interventions will reduce the risk of dialysis events.

